# Supplementary material for: Artesunate, EDTA, and colistin work synergistically against MCR-negative and -positive colistin-resistant Salmonella
Source: eLife. 2025 Feb 7;13:RP99130. doi: 10.7554/eLife.99130 (PMC11805504; doi:10.7554/eLife.99130)
Supplement: Supplementary file 1. — (a) Sequences of primers used in this study. (b) The antibacterial activities of COL, AS, and EDTA against the tested strains after single and double combinations. (c) The antibacterial activities of COL against the tested strains after single and triple combinations. (d) The MICs of different antimicrobial drugs against the S16 and S30. [file elife-99130-supp1.docx]

**Supplementary file 1**

**Artesunate, EDTA and colistin work synergistically against MCR-negative and -positive colistin-resistant *Salmonella***

Yajun Zhai^1^†, Peiyi Liu^1^†, Xueqin Hu^1^†, Changjian Fan^1^, Xiaodie Cui^1^, Qibiao He^1^, Dandan He^1^, Xiaoyuan Ma^1^, Gongzheng Hu^1^**^*^**, Yajun Zhai^1^**^*^**

^1^Department of Pharmacology and Toxicology, College of Veterinary Medicine, Henan Agricultural University, Zhengzhou, Henan 450046, China

† The authors contributed equally as the first author.

**^*^**Corresponding authors Address: College of Veterinary Medicine, Henan Agricultural University, Zhengzhou 450002, People’s Republic of China;

E-mail address: [zyj90518@126.com](mailto:zyj90518@126.com) (Yajun Zhai), [yaolilab@126.com](mailto:yaolilab@126.com) (Gongzheng Hu)

Running title: Synergy of artesunate, EDTA and colistin

**Supplementary file 1a. Sequences of primers used in this study**

| **Primer** | **Sequence (5’ → 3’)** | **References** |
| --- | --- | --- |
| *cheA* – F | AATCTCGAGGTGAGCATGGATATTAGCGA | This study |
| *cheA* – R | AATGAATTCTCAGGCGGCTGTGATCGCCA |  |
| *cheY* – F | ACACTCGAGATGGCGGATAAAGAGCTTAA | This study |
| *cheY* – R | ACAGAATTCTCACATGCCCAGTTTCTCAA |  |
| *STMDT2_34621* - F | GGCCTCGAGATGAAAAATATCAAAGTCATCAC | This study |
| *STMDT2_34621* - R | GGCGAATTCTTAGAAGCTTTCCCAGTTCG |  |
| *aer* - F | AATCTCGAGATGTCTTCTCATCCCTACGT | This study |
| *aer* - R | GGCCTGCAGTTAATGCAGTACCGTGA |  |
| *fliD* - F | GGCCTCGAGATGGCTTCAATTTCATCATT | This study |
| *fliD* - R | AACCTGCAGTCAGGACTTGTTCATAGCT |  |
| *fliT* - F | AATCTCGAGATGACCTCAACCGTGGA | This study |
| *fliT* - R | AATGAATTCTTATGAGGCGCCAGGCG |  |
| *oppuBB* - F | GGCCTCGAGATGGATACGATACATTATATG | This study |
| *oppuBB* - R | AATGAATTCTTATCGTATCCCCTTCGGTG |  |
| *gltI* - F | GGCCTCGAGATGATAAAGGAGTTGGATAT | This study |
| *gltI* - R | GGCGAATTCTTAGTTAAGCGCTTTATCATTC |  |
| *dppB* - F | AATCTCGAGATGTTGCAGTTCATTCTCCG | This study |
| *dppB* - R | GGCGAATTCTTACTTCTTATGCCGAATACGC |  |
| *dppC* - F | AGGCTCGAGATGTCACAGGTTACTGAAAA | This study |
| *dppC* - R | AGGGAATTCTTACTGCTTCAGTTTGGGATC |  |
| *spvD* - F | AATCTCGAGATGAGAGTTTCTGGTAGTGC | This study |
| *spvD* - R | GGCGAATTCTCAATCGTGTTTTTCATCAT |  |
| *mcr*-*1* - qF | TGCTCCAAAATGCCCTACAGACC | Yi *et al*, 2022 |
| *mcr*-*1* - qR | TGCCCCAAGTCGGATAATCCAC |  |
| 16S rRNA - qF | TGTCGTCAGCTCGTGTTGTG | Yi *et al*, 2022 |
| 16S rRNA - qR | ATCCCCACCTTCCTCCAGTT |  |

**Supplementary file 1b. The antibacterial activities of COL, AS and EDTA against the tested strains after single and double combinations**

| Strains | *mcr-1* | MICs (mg/L) | | | | | | | | | | |
| --- | --- | --- | --- | --- | --- | --- | --- | --- | --- | --- | --- | --- |
|  |  | Alone | | | COL + AS | | | | COL + EDTA | | | |
|  |  | COL | AS | EDTA | 1/4  AS | 1/8  AS | 1/16  AS | **Fold**  **change** | 1/4  EDTA | 1/8  EDTA | 1/16  EDTA | **Fold**  **change** |
| S29 | + | 2 | 1250 | 125 | 0.015 | 0.125 | 0.5 | **4-133** | 1 | 1 | 2 | **0-2** |
| S31 | + | 4 | 1250 | 125 | 0.25 | 0.5 | 2 | **2-16** | 2 | 2 | 2 | **0** |
| S23 | + | 4 | 1250 | 125 | 0.25 | 0.5 | 1 | **4-16** | 2 | 2 | 2 | **0** |
| S93 | + | 4 | 1250 | 125 | 0.25 | 2 | 2 | **2-16** | 2 | 2 | 2 | **0** |

**Supplementary file 1c. The antibacterial activities of COL against the tested strains after single and triple combinations**

| Strains | MICs (mg/L) | | | | | | | | | **Fold change** |
| --- | --- | --- | --- | --- | --- | --- | --- | --- | --- | --- |
|  | COL  alone | COL + 1/4 AS +EDTA | | | | COL + 1/8 AS +EDTA | | | |  |
|  |  | 1/4  EDTA | 1/8  EDTA | 1/16  EDTA | 1/32  EDTA | 1/4  EDTA | 1/8  EDTA | 1/16  EDTA | 1/23  EDTA |  |
| S29 | 2 | 0.00003 | 0.001 | 0.03 | 0.0625 | 0.008 | 0.03 | 0.125 | 0.125 | **16-66667** |
| S31 | 4 | 0.03 | 0.25 | 0.25 | 0.5 | 0.125 | 0.25 | 0.5 | 0.5 | **8-133** |
| S23 | 4 | 0.0625 | 0.125 | 0.125 | 0.25 | 0.125 | 0.25 | 0.5 | 0.5 | **8-64** |
| S93 | 4 | 0.03 | 0.0625 | 0.125 | 0.25 | 0.25 | 0.5 | 0.5 | 2 | **2-133** |

**Supplementary file 1d. The MICs of different antimicrobial drugs against the S16 and S30**

| Drug | FFC | ENR | DOX | AMP | AMK | FOS | CAZ | CRO | CTX | TGC |
| --- | --- | --- | --- | --- | --- | --- | --- | --- | --- | --- |
| Breakpoint | ≥16 | 2 | ≥16 | ≥32 | ≥16 | ≥256 | ≥16 | ≥4 | ≥4 | ≥4 |
| S16 | 2 | ＜0.5 | 2 | 512 | ＜0.5 | 2 | ＜0.5 | ＜0.5 | ＜0.5 | 4 |
| S30 | 128 | 2 | 32 | ＞512 | 1 | 8 | ＜0.5 | ＜0.5 | ＜0.5 | 4 |

Note: FFC: Flufenicol; ENR: Enrofloxacin; DOX: Doxycycline; AMP: Ampicillin; AMK: Amikacin; FOX: Fosfomycin; CAZ: ceftazidime; CRO: Ceftriaxone; CTX:Cefotaxime; TGC: Tigecycline.
